# Beyond IQ: The Importance of Metacognition for the Promotion of Global Wellbeing

**DOI:** 10.3390/jintelligence9040054

**Published:** 2021-11-18

**Authors:** Lav R. Varshney, Aron K. Barbey

**Affiliations:** 1 Department of Electrical and Computer Engineering, University of Illinois at Urbana-Champaign, Urbana, IL 61801, USA; varshney@illinois.edu; 2 Beckman Institute for Advanced Science and Technology, University of Illinois at Urbana-Champaign, Champaign, IL 61801, USA; 3 Department of Psychology, University of Illinois at Urbana-Champaign, Champaign, IL 61801, USA

**Keywords:** collective intelligence, metacognition, wellbeing

## Abstract

Global policy makers increasingly adopt subjective wellbeing as a framework within which to measure and address human development challenges, including policies to mitigate consequential societal problems. In this review, we take a systems-level perspective to assemble evidence from studies of wellbeing, of collective intelligence, and of metacognition and argue for a virtuous cycle for health promotion in which the increased collective intelligence of groups: (1) enhances the ability of such groups to address consequential societal problems; (2) promotes the wellbeing of societies and the individual wellbeing of people within groups; and, finally, (3) enables prosocial actions that further promote collective problem-solving and global wellbeing. Notably, evidence demonstrates that effective collaboration and teamwork largely depend on social skills for metacognitive awareness—the capacity to evaluate and control our own mental processes in the service of social problem-solving. Yet, despite their importance, metacognitive skills may not be well-captured by measures of general intelligence. These skills have instead been the focus of decades of research in the psychology of human judgment and decision-making. This literature provides well-validated tests of metacognitive awareness and demonstrates that the capacity to use analysis and deliberation to evaluate intuitive responses is an important source of individual differences in decision-making. Research in network neuroscience further elucidates the topology and dynamics of brain networks that enable metacognitive awareness, providing key targets for intervention. As such, we further discuss emerging scientific interventions to enhance metacognitive skills (e.g., based on mindfulness meditation, and physical activity and aerobic fitness), and how such interventions may catalyze the virtuous cycle to improve collective intelligence, societal problem-solving, and global wellbeing.

## 1. Collective Intelligence, Metacognition, and Wellbeing 

Collective intelligence, metacognition, and wellbeing are three constructs that have developed largely independently from one another in psychology and allied fields, which we review here. Yet, emerging evidence indicates there are potentially powerful relationships among them that can be leveraged to catalyze collective problem-solving at a systems level to address consequential societal troubles such as the coronavirus pandemic, systemic racism, and poverty. In particular, social problem-solving is largely driven by social engagement (Mao 2016) and the collective intelligence of a community (Tovey 2015; Malone and Woolley 2020), which itself depends on effective collaboration and teamwork due to social skills from metacognitive awareness (Gupta and Woolley 2020). Further, social problem-solving can equitably promote public wellbeing, which may be necessary for promoting individual wellbeing (Lambert et al. 2020) and in turn lead to people that are more socially engaged (Mehl et al. 2010; Richards and Huppert 2011). Here we argue that scientific interventions to enhance metacognitive awareness may improve collective intelligence, social problem-solving, and global wellbeing.

Global policy makers increasingly adopt subjective wellbeing as a framework within which to measure and address human development challenges, extending their traditional focus from material wellbeing and economic development to include the impact policies have on how people think and feel about their lives (Dolan and White 2007). Further, there is growing agreement among scientists, economists, and policy experts that the gross domestic product (GDP) should no longer be a primary performance indicator for national prosperity. The World Happiness Report (WHR) has garnered attention for its national happiness rankings; it assesses wellbeing using the Cantril Self-Anchoring Striving Scale (Cantril 1965), also called Cantril’s Ladder, which rates respondents on their current and future perceived quality or satisfaction with life. Yet, as captured in the United Nations 2030 Sustainable Development Goals (SDGs), an emerging global agenda for development is recognizing the coupling of social, environmental, and economic dynamics and in particular, the interconnectedness of several dimensions of human and ecosystem wellbeing (Fioramonti et al. 2019). Moreover, the science of wellbeing is starting to reflect a richer view of wellbeing than life satisfaction alone by including hedonic and eudaemonic facets of wellbeing, social wellbeing, as well as the role of culture, community, nature, and governance (Lambert et al. 2020). Assessment tools beyond Cantril’s Ladder that draw not just on psychology, but also neighboring fields such as organizational design, health, education, and economics, are being developed for this broader conception of wellbeing. 

Given that subjective wellbeing in this broader conception is not only an individual consideration but is intertwined with social and environmental life, it follows that equitably promoting public wellbeing is necessary for promoting individual wellbeing. Quite interestingly, equality not just of income but also of life satisfaction is associated with higher average subjective wellbeing in countries around the world (Diener and Tay 2015). Further, the positive impact of income on subjective wellbeing is greater in more equal societies (Ng and Diener 2019). Due to the positively correlated relationships between life satisfaction and subjective wellbeing as well as between income and subjective wellbeing, we can therefore infer that subjective wellbeing is not a conserved, extensive quantity like energy or mass, but actually increases in total as it is equalized in societies. This indicates that redistribution is not a zero-sum activity. 

It further follows that public wellbeing is inextricably linked with consequential societal problems such as the coronavirus pandemic, systemic racism, and poverty. Public wellbeing can be promoted through collective action on social determinants of wellbeing (Fisher 2019), especially since a key driver of wellbeing is the capacity to distribute and share the cognitive, social, and economic demands of life, providing a powerful mechanism for mitigating loss and the effects of adversity. 

Evidence demonstrates that people with greater individual wellbeing are more likely to show compassion and empathy (Shanafelt et al. 2005; Nelson 2009; Rand et al. 2015), make ethical decisions (James and Chymis 2004; Fernando and Chowdhury 2010), live healthier and longer lives (Wiest et al. 2011; Boehm and Kubzansky 2012), and perhaps most importantly for our discussion, be socially engaged (Mehl et al. 2010; Richards and Huppert 2011) and perform prosocial behavior (Son and Wilson 2012; Yang et al. 2017). 

At the same time, recent findings suggest that addressing consequential societal problems and enhancing prosperity are largely driven by social problem-solving and the collective intelligence of a community (Tovey 2015; Malone and Woolley 2020), rather than the individual intelligence of community members. Contemporary methods to enhance collective intelligence have focused on the distribution and processing of knowledge within a social network, as measured by transactive memory and attentional systems (Gupta and Woolley 2020). Notably, the collective intelligence of a group is strongly associated with abilities such as emotional intelligence and theory of mind of group members (Woolley et al. 2010). Further, effective collaboration and teamwork largely depend on social skills for metacognitive awareness (Gupta and Woolley 2020), the capacity to evaluate and control our own mental processes in the service of social problem-solving.

To summarize the logic that emerges from our review, we see a virtuous cycle for health promotion in which the increased collective intelligence of groups: (1) enhances the ability of such groups to address consequential societal problems; (2) promotes the wellbeing of societies and the individual wellbeing of people within groups; and, finally, (3) enables prosocial actions that further promote collective problem-solving and global wellbeing.

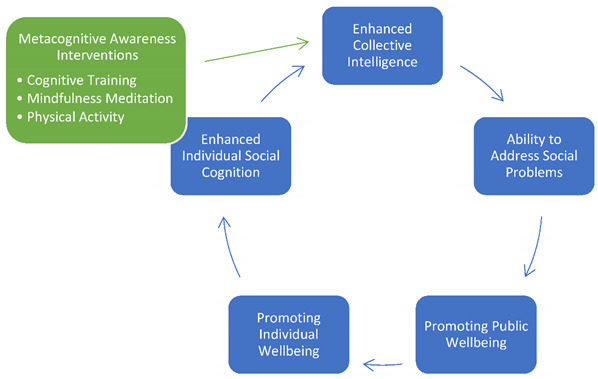



Is it possible to accelerate this virtuous cycle and enhance global wellbeing through science-based interventions that focus on particular metacognitive abilities, which have known neural correlates in brain networks? 

## 2. Construct Validity

By analogy with the individual intelligence factor *g*, Woolley et al. (2010) defined a group’s *collective intelligence* (*c*) as the general ability of the group to perform a wide variety of tasks. From a psychometric perspective, *c* emerges when the ability of a group to perform one task is correlated with that group’s ability to perform a wide range of other tasks. This kind of collective intelligence is a property of the group itself, not just the individuals in it. Although the individual intelligence factors of group members may play a role in collective intelligence (Bates and Gupta 2017), a meta-analysis that examined 22 studies, with 5279 individuals comprising 1356 groups, showed that *c* is predicted by the proportion of women in the group, mediated by average social perceptiveness of group members, and that it is validated in the sense of predicting performance on various out-of-sample criterion tasks (Riedl et al. 2021). Yet, *c* is not nearly as well-validated as *g* (Coyle 2021).

*Metacognitive awareness*, with a key dimension of metacognitive knowledge, is defined as beliefs about one’s own mental states and processes as well as beliefs about those of other people (Jost et al. 1998). Aptitude in these specific metacognitive skills is the focus of our analysis. There is some agreement on the general theoretical structure of metacognition, which has informed the development of the Metacognitive Awareness Inventory (MAI), a commonly used instrument for its measurement (Schraw and Dennison 1994). Although self-report instruments such as MAI may raise validity concerns, it is widely used in both research and practice; further refinements show between-group and time invariance (Harrison and Vallin 2018). 

As noted above, *subjective wellbeing* is defined as how people experience and evaluate their lives, usually measured through questionnaires that have been well-validated using large-scale studies (Nima et al. 2020). An example instrument is the Satisfaction With Life Scale (Pavot and Diener 2008), which has been validated and widely used. Wellbeing is increasingly being constructed to also include hedonic and eudaemonic facets of wellbeing, social wellbeing, as well as the role of culture, community, nature, and governance (Lambert et al. 2020). This validity of this broader construct of subjective wellbeing is still under investigation (Pancheva et al. 2021).

## 3. Interventions to Improve Wellbeing via Improved Metacognitive Skills

Despite their importance for collective intelligence (Gupta and Woolley 2020) and wellbeing (Kiaei and Reio 2014), metacognitive skills are not typically captured by measures of general intelligence (and are in fact weakly associated with intelligence (Ohtani and Hisasaka 2018)). These skills have instead been the focus of research in educational psychology and the psychology of human judgment and decision-making (Koriat 2015). This literature provides well-validated tests of metacognitive awareness (Schraw and Dennison 1994) and demonstrates that the capacity to use analysis and deliberation to evaluate intuitive responses is an important source of individual differences in decision-making (Kleitman et al. 2019). Moreover, this literature demonstrates that metacognitive training aids decision-making (Batha and Carroll 2007). 

Research in network neuroscience further elucidates the topology and dynamics of brain networks that enable metacognitive awareness, providing key targets for intervention (Paul et al. 2015). Evidence indicates that the precuneus has differential connectivity within the default mode network of the brain across individuals’ lifespans (Yang et al. 2014) and has specialized roles in self-related cognition and awareness (Philippi et al. 2012; Whitfield-Gabrieli et al. 2011). These attributes of the precuneus suggest it may be linked with over/underconfidence in decision-making or other metacognitive abilities. In our recent work, we collected resting-state fMRI data (*n* = 304) to perform connectome-wide association to characterize individual differences in decision-making competence, identifying regions within the frontal, parietal, temporal, and occipital cortex that demonstrated significant associations (Talukdar et al. 2018). In assessing whether functional interactions between brain regions sensitive to decision-making competence and particular intrinsic connectivity networks (ICNs) were predictive of specific facets of decision-making, we further found individual differences in specific facets of decision-making competence are mediated by ICNs that support executive, social, and perceptual processes. More broadly, these findings motivate an integrative framework for understanding the neural basis of individual differences in decision-making competence (Talukdar et al. 2018). 

Research in cognitive psychology has investigated several pathways to improve metacognitive skills including skill-based cognitive training and mindfulness meditation. A large empirical literature on the efficacy of cognitive training programs, involving the guided practice of specific cognitive tests to enhance executive functions and decision-making (Zwilling et al. 2019), indicates that training of executive functions can promote specific cognitive skills (e.g., inhibition, interference control, and working memory) but typically does not generalize to improvements beyond the trained tasks (Au et al. 2014; Simons et al. 2016; Soveri et al. 2017; Butler et al. 2018). Although still very much an active area of research, transfer of metacognitive training may be more effective (Jones et al. 2020; Schaeffner et al. 2021) than transfer of cognitive training, which demonstrates limited generalization (Sala and Gobet 2017, 2019).

Accumulating evidence indicates that the neural mechanisms underlying improvements in executive function from cognitive training are due to changes in functional connectivity, such as increased neural synchrony between frontal and parietal regions (Constantinidis and Klingberg 2016). Potential drivers of this increased functional connectivity include stronger synaptic connections (Gibson et al. 2014), increased myelination of the connecting axons (Yeung et al. 2014), or increased release rate of dopamine (Backman et al. 2011). The central role of executive functions in metacognitive skills (del Missier et al. 2012) motivates the application of interventions from cognitive psychology—which are designed to target the cognitive and neural mechanisms underlying executive functions—to enhance metacognitive awareness. This represents a promising direction for future interventions and studies that aim to confer benefits that generalize beyond the training context. 

An emerging area of research in psychology investigates the beneficial effects of mindfulness meditation on metacognition. Evidence indicates that mindfulness training cultivates moment-to-moment awareness of the self and environment (Wallace 2006) and to this extent, mindfulness training heightens metacognition (Austin 1998). This provides a pathway to explain robust experimental findings on the impact of mindfulness training on metacognition (Zeidan et al. 2010; Solem et al. 2015). Neuroscience evidence further demonstrates that mindfulness meditation induces changes in functional brain connectivity within the ventral attention network (Brefczynski-Lewis et al. 2007), dorsal anterior cingulate (Tang et al. 2009, 2010), medial frontal cortex (Hölzel et al. 2007), temporal parietal junction, and pons (Hölzel et al. 2011). These findings suggest that mindfulness meditation enhances the cognitive and neural mechanisms of executive function (which is an independent but highly overlapping construct with metacognitive awareness (Fernandez-Duque et al. 2000)) and motivates applications to metacognition. 

A complementary literature in health psychology investigates the efficacy of moderate intensity physical activity and aerobic fitness training to enhance executive functions (for reviews, see Hillman et al. 2008; Voss et al. 2011; Guiney and Machado 2013). A recent meta-analysis demonstrates that physical activity and aerobic fitness enhances executive functions, observing an effect size gain of 0.34 across 36 studies (Northey et al. 2017). Neuroscience evidence further indicates that the observed improvements in executive functions are linked to the effects of physical activity and aerobic fitness on brain structure and function. A growing body of evidence indicates that aerobic fitness promotes efficient functional connectivity within brain networks for executive function, primarily within the fronto-parietal network (Colcombe et al. 2004; Voss et al. 2010a, 2011). For example, Voss et al. (2010b) demonstrated that a 1-year walking intervention was associated with increased functional connectivity within the fronto-parietal network of healthy older adults. Taken together, evidence from health psychology and neuroscience demonstrates that aerobic fitness improves executive functions, suggesting that physical fitness may have beneficial effects on associated skills for metacognitive awareness.

The reviewed findings support the efficacy of modern interventions from psychology to enhance metacognitive awareness and associated executive functions. Although unimodal approaches to intervention represent the most commonly applied method, an emerging body of evidence examines the efficacy of multi-modal interventions designed to leverage the beneficial effects of multiple intervention modalities. For example, recent evidence demonstrates that multi-modal cognitive and physical fitness training produces greater improvements in executive functions compared to unimodal training (Fabre et al. 2002; Oswald et al. 2006; Ward et al. 2017; Daugherty et al. 2018; Zwilling et al. 2019). A meta-analysis investigating the combined effects of cognitive and physical fitness training across 20 studies concluded that multi-modal training delivers synergistic effects that enhance performance more than physical fitness training or cognitive training alone (Lauenroth et al. 2016). Although the mechanisms underlying the beneficial effects of multi-modal training are still under investigation, animal models suggest that both cognitive and physical fitness training may promote neural plasticity and stimulate neurogenesis (Fabel 2009). These findings support the efficacy of multi-modal interventions—providing evidence that this approach can enhance performance on tests of executive function and further motivating their potential for the promotion of metacognitive awareness, collective intelligence, and subjective wellbeing. 

## 4. Summary

In this short review article, we brought together findings from psychology, cognitive neuroscience, and the social science of intelligence to argue for a virtual cycle of global wellbeing that can be accelerated via interventions to enhance individual metacognition (due its central role in collective intelligence). Notably, there are methods from cognitive training, mindfulness meditation, and kinesiology that have been demonstrated to enhance metacognitive awareness, which can be deployed as large-scale interventions. As noted, promoting global population-level and individual wellbeing via improved metacognition will be fundamentally intertwined with addressing vexing societal problems such as inequity.

## Data Availability

Not applicable.
